# The entropy production for thermal operations

**DOI:** 10.1038/s41598-020-66416-9

**Published:** 2020-06-16

**Authors:** H. Dolatkhah, S. Salimi, A. S. Khorashad, S. Haseli

**Affiliations:** 1 Department of Physics, University of Kurdistan, P. O. Box: 66177-15175 Sanandaj, Iran; 2 Faculty of Physics, Urmia University of Technology, Urmia, Iran

**Keywords:** Quantum information, Thermodynamics

## Abstract

According to the first and second laws of thermodynamics and the definitions of work and heat, microscopic expressions for the non-equilibrium entropy production have been achieved. Recently, a redefinition of heat has been presented in [Nature Communications volume 8, Article number: 2180 (2017)]. Since thermal operations play an important role in the resource theory of thermodynamics, it would be very interesting to find out the effect of the above-mentioned definition on the expression of the entropy production for these kind of operations. This is one of the aims of the present paper. Using the new definition of heat, it is shown that the entropy production is the same as the mutual information between a system and a bath both for thermal operations and, if the system-bath initial state is factorized, for entropy-preserving operations. It is also discussed that how one can recognize the type of the correlation between a system and a bath through knowledge of the initial state of the system only. It is shown that if the initial state of a system is diagonal in the energy basis, the thermal operations cannot create a quantum correlation between the system and the bath, however, if the system initial state is coherent Gibbs state, there cannot be classical correlation due to the thermal operations.

## Introduction

Recently, study on thermodynamic behavior of quantum mechanical systems has attracted much attention. In fact, providing a clear understanding about the fundamental concepts such as work and heat, and obtaining a deep knowledge of thermodynamics laws in quantum world, have been the main topic of many researches^1–11^. To understand the foundations of quantum thermodynamics, one can consider it as a resource theory. There are different models for the resource theories of thermodynamics^12–14^, which vary mostly on the set of allowed operations. One of the most important models is the resource theory of thermal operations (TOs). TOs were introduced in ref. ^15^ and applied later in refs. ^16–19^.

The set of TOs, {*ε*_*T*_}, consists of all maps acting on the state of a system as^15–17^:

1 $${\rho {\prime} }_{S}={\varepsilon }_{T}({\rho }_{S})=T{r}_{B}({\rho {\prime} }_{SB}),$$

in which $T{r}_{B}$ is partial trace over the bath, and ${\rho }_{SB}^{\text{'}}$ is the state of the composite system after the evolution, i.e.,

$${\rho }_{SB}^{\text{'}}={U}_{SB}({\rho }_{S}\otimes {\rho }_{B}^{eq}){U}_{SB}^{\dagger },$$

where

1. *U*_*SB*_ is an energy-preserving unitary operator applied to the system and the bath satisfying
  2 $$[{U}_{SB},{H}_{S}+{H}_{B}]=0,$$
  here, *H*_*S*_ and *H*_*B*_ are Hamiltonians of the system and the bath, respectively.
2. ${\rho }_{B}^{eq}$ is a *thermal* state of the bath at some fixed temperature,

3 $${\rho }_{B}^{eq}=\frac{{e}^{-\beta {H}_{B}}}{{Z}_{B}},$$

where $\beta =\frac{1}{{k}_{B}T}$ (throughout the paper it is assumed that *k*_*B*_ = 1), and ${Z}_{B}=tr({e}^{-\beta {H}_{B}})$ is known as the partition function.

There are two important properties for TOs which are^20,21^:

1. They have time translation symmetry,
  4 $${\varepsilon }_{T}({e}^{-i{H}_{S}t}{\rho }_{S}{e}^{i{H}_{S}t})={e}^{-i{H}_{S}t}{\varepsilon }_{T}({\rho }_{S}){e}^{i{H}_{S}t},$$
2. They preserve the thermal state,

5 $${\varepsilon }_{T}({\rho }_{S}^{eq})={\rho }_{S}^{eq}\mathrm{}.$$

Regarding the first law of thermodynamics and this fact that the thermal bath is an incoherent mixture of energy states, Eq. (4) indicates conservation of energy. Equation (5) expresses that it is impossible to change a thermal state without doing any work. This means that there cannot exist any machine working in a cycle and converting thermal energy into work completely. This is actually the physical meaning of the second law of thermodynamics.

The conservation of energy in thermodynamic systems is the topic of the first law of thermodynamics, which states that every increase in the internal energy of a system is due to the following two ways: (a) the work performed on the system and/or (b) the heat absorbed by the system. Irreversible processes are described by the second law of thermodynamics. According to this law, the entropy production is always non-negative; it is zero only when the system and the environment are in thermal equilibrium. Regarding the first and second laws of thermodynamics, one can derive microscopic expressions for the non-equilibrium entropy production in quantum systems^22–27,45^.

Recently, a new definition of heat has been provided in which the authors introduced heat by properly referring to the information flow and thereby restoring Landauer’s erasure principle^28^. Here, the effect of the definition on the expression of the entropy production for TOs is determined. Since microscopic expression of the non-equilibrium entropy production depends on the definition of heat, one can expect that any new definition of it might change the expression of the entropy production. It is also shown that TOs cannot generate quantum correlation from incoherent input states, however, if the initial state of a system is a coherent Gibbs state, correlation between the system and the bath is quantum correlation.

In the following, firstly, free energy definition and heat definitions are presented. Secondly, using the heat definitions, the corresponding expressions of non-equilibrium entropy production are obtained. Finally, the role of quantum coherence in the entropy production for TOs is studied.

## Preliminary

The non-equilibrium free energy for a system in a state *ρ*_*S*_ with Hamiltonian *H*_*S*_, which interacts with a thermal bath at temperature *T*, is defined as:

6 $$F({\rho }_{S})={E}_{S}-TS({\rho }_{S}),$$

where *E*_*S*_ = *tr*(*H*_*S*_*ρ*_*S*_) is internal energy and *S*(*ρ*_*S*_) = −*tr*(*ρ*_*S*_ln(*ρ*_*S*_)) is the von Neumann entropy of the system. If one uses ${H}_{S}=-\frac{1}{\beta }(ln({\rho }_{S}^{eq})+ln({Z}_{S}))$, the non-equilibrium free energy can be written as

7 $$F({\rho }_{S})={F}_{eq}+TS({\rho }_{S}||{\rho }_{S}^{eq}),$$

where ${F}_{eq}=\frac{-1}{\beta }ln({Z}_{S})$ is the free energy in thermal equilibrium, and $S({\rho }_{S}||{\rho }_{S}^{eq})$ is the relative entropy. It is worth mentioning that $F({\rho }_{S})\ge {F}_{eq}$ due to the non-negativity of the relative entropy.

Usually, heat is defined as the change in the internal energy of the bath^29,31^,

8 $$\Delta \bar{Q}=-\,\Delta {E}_{B}\mathrm{}.$$

Although many researchers have used this definition in their works^24,29,31^, it has been recently shown that it is not a perfect definition for heat^28^. To provide a more proper definition, one can assume that there is a thermal bath whose state is initially given by a thermal state ${\rho }_{B}^{eq}$ and it is subject to Hamiltonian *H*_*B*_ at temperature *T*. In a process, where the bath state ${\rho }_{B}^{eq}$ transforms to ${\rho }_{B}^{\text{'}}$ under the condition that the Hamiltonian *H*_*B*_ is remained unchanged, heat is defined as^28^

9 $$\Delta Q=-\,(\Delta {E}_{B}-\Delta {F}_{B})=\frac{-1}{\beta }\Delta {S}_{B},$$

where $\Delta {F}_{B}=F({\rho }_{B}^{\text{'}})-F({\rho }_{B}^{eq})$ is the change in the free energy of the bath which is stored in the bath as the extractable work. $\Delta {S}_{B}=S({\rho }_{B}^{\text{'}})-S({\rho }_{B}^{eq})$ is the change in the von Neumann entropy of the bath due to the state transformation. Since in this approach heat is expressed in terms of the entropy difference of the bath, one can say that there is an explicit relation between heat and information flow to or from the bath. This is consistent with Landauer’s erasure principle^30^. According to Eq. (9), heat is also responsible for the change in the internal energy of the bath. However, the bath internal energy can be varied through other form of energy flow on the condition that entropy is preserved. This form of energy flow is stored as extractable work. Comparing Eq. (8) with Eq. (9), one has

10 $$\Delta Q=\Delta \bar{Q}+\Delta {F}_{B}\mathrm{}.$$

Since Δ*F*_*B*_ is always non-negative (due to this fact that the initial state of the bath is thermal and the free energy for this state has its minimum value), one comes to

11 $$\Delta Q\ge \Delta \bar{Q}\mathrm{}.$$

If the bath deviates from thermal equilibrium by small variation through a TO, ${\rho }_{B}^{\text{'}} \sim {\rho }_{B}^{eq}+\varepsilon$, one will have $\Delta {F}_{B}=TS({\rho }_{B}^{{\prime} }||{\rho }_{B}^{eq})\sim {\varepsilon }^{2}$ which goes to zero in the limit of large bath. Therefore, both definitions are consistent.

## New entropy production for thermal operations

When a system experiences a dynamical process, the change in its entropy, Δ*S*_*S*_, includes a reversible and an irreversible contribution. The reversible contribution is due to the heat flow, which can be addressed as the entropy flow $\Delta {S}^{rev}=\beta \Delta Q$, and the irreversible one is called entropy production Δ*S*^*irr*^. Therefore, one can write

12 $$\Delta {S}_{S}=\Delta {S}^{irr}+\Delta {S}^{rev}\mathrm{}.$$

Regarding the usual definition of heat, Eq. (8), and the total change in the entropy of a system, Eq. (12), one can obtain the entropy production for TOs,

13 $$\Delta {\bar{S}}^{irr}=-\,\beta \Delta {F}_{S}=S({\rho }_{S}||{\rho }_{S}^{eq})-S({\rho }_{S}^{\text{'}}||{\rho }_{S}^{eq}),$$

which is the familiar form of the entropy production for TOs^23,35^ (see Methods for more detail). $\Delta {\bar{S}}^{irr}$ is always non-negative due to the contraction of the quantum relative entropy^32^. As a result

14 $$\Delta {F}_{S}\le 0,$$

meaning that free energy of the system is decreasing under TOs^18,20^. According to the definition of TO, Eq. (13) can be written as^24,25^:

15 $$\Delta {\bar{S}}^{irr}=S({\rho }_{B}^{\text{'}}||{\rho }_{B}^{eq})+I({\rho }_{SB}^{\text{'}}),$$

where $I({\rho }_{SB}^{\text{'}})=S({\rho }_{S}^{\text{'}})-S({\rho }_{B}^{\text{'}})-S({\rho }_{SB}^{\text{'}})$ is the mutual information between the system and the bath. Equation (15) shows that the change in the state of the bath along with the mutual information is responsible for the entropy production variation.

Now, let us obtain an expression for the entropy production for TOs based on the new definition of heat presented in the previous section. If one uses Eq. (9) in Eq. (12), one obtains

16 $$\Delta {S}^{irr}=-\,\beta (\Delta {F}_{S}+\Delta {F}_{B})=S({\rho }_{S}||{\rho }_{S}^{eq})-S({\rho }_{S}^{\text{'}}||{\rho }_{S}^{eq})-S({\rho }_{B}^{\text{'}}||{\rho }_{B}^{eq}),$$

which is the new form of the entropy production for TOs, more detail can be found in Methods. Considering the definition of TO, Eq. (16) can be rewritten as

17 $$\Delta {S}^{irr}=I({\rho }_{SB}^{\text{'}}).$$

Since $I({\rho }_{SB}^{\text{'}})$ is always non-negative, one comes to Δ*S*^*irr*^ ≥ 0. As can be seen in Eq. (17), it is just the mutual information which determines the entropy production; there is no term showing the change of bath state. It is important to note that for entropy-preserving operations^28^ if the initial state of the composite system *SB* is factorized, Eq. (17) is also true (see Methods).

Regarding the non-negativity of Δ*S*^*irr*^ together with Eq. (16), one obtains

18 $$\Delta {F}_{S}+\Delta {F}_{B}\le \mathrm{0,}$$

which means that sum of the system and the bath free energies is decreasing under TOs. Equation (18) can be rewritten as

19 $$\Delta {F}_{S}\le -\,\Delta {F}_{B},$$

which introduces an upper bound for Δ*F*_*S*_. As can be seen, unlike Eq. (14), this upper bound depends on the change in the free energy of the bath. Also, it is tighter than Eq. (14), due to the positivity of Δ*F*_*B*_.

## Quantum coherence and entropy production

Quantum coherence is one of the most important concepts in quantum physics. Recently, the role of the quantum coherence in determining the behavior of the entropy production has been investigated^35^. It has been shown that the non-equilibrium free energy of a system can be written as^20,35,36^:

20 $$F({\rho }_{S})={F}_{eq}+TS({\Delta }_{{H}_{S}}({\rho }_{S})||{\rho }_{S}^{eq})+TC({\rho }_{S}),$$

where $S\mathrm{(}.||\mathrm{.)}$ is quantum relative entropy and *C*(*ρ*_*S*_) is relative entropy of coherence^33,34^,

21 $$C({\rho }_{S})=S({\Delta }_{{H}_{S}}({\rho }_{S}))-S({\rho }_{S}),$$

where

$${\Delta }_{{H}_{S}}({\rho }_{S})=\sum _{i}\langle {E}_{i}|{\rho }_{S}|{E}_{i}\rangle |{E}_{i}\rangle \langle {E}_{i}|$$

is a dephasing map acting on density matrix *ρ*_*S*_ and removing all coherences in the energy basis $(\{|{E}_{i}\rangle \})$, (see Methods). According to Eq. (20), the entropy production is divided into two parts: classical and quantum^35,36^,

22 $$\Delta {\bar{S}}^{irr}=\Delta {\bar{S}}_{C}^{irr}+\Delta {\bar{S}}_{Q}^{irr},$$

where

23 $$\Delta {\bar{S}}_{C}^{irr}=S({\Delta }_{{H}_{S}}({\rho }_{S})||{\rho }_{S}^{eq})-S({\Delta }_{{H}_{S}}({\rho }_{S}^{\text{'}})||{\rho }_{S}^{eq})$$

is the classical part, and

24 $$\Delta {\bar{S}}_{Q}^{irr}=C({\rho }_{S})-C({\rho }_{S}^{\text{'}})$$

is the quantum one. Since the diagonal elements of a density matrix are transformed independently of the off-diagonal ones in state-to-state transformation under TOs, $\Delta {\bar{S}}_{C}^{irr}$ is non-negative. Also, $\Delta {\bar{S}}_{Q}^{irr}$ is positive because TO is incoherent^33^. A quantum operation is coherence-preserving if and only if it is unitary and incoherent^37^. Therefore, the unitary operator *U*_*SB*_, introduced in the definition of TO, is a coherence-preserving operator. Thus, the total coherence of system+bath remains unchanged under the operation of *U*_*SB*_,

25 $$C({\rho }_{SB}^{\text{'}})=C({U}_{SB}({\rho }_{S}\otimes {\rho }_{B}^{eq}){U}_{SB}^{\dagger })=C({\rho }_{S}\otimes {\rho }_{B}^{eq})=C({\rho }_{S}),$$

which is due to this fact that the relative entropy of coherence is additive on tensor product states and ${\rho }_{B}^{eq}$ is an incoherent state. Substituting Eq. (25) into Eq. (24), one obtains^35^

26 $$\Delta {\bar{S}}_{Q}^{irr}={C}_{cc}({\rho }_{SB}^{\text{'}})+C({\rho }_{B}^{\text{'}}),$$

where ${C}_{cc}({\rho }_{SB}^{\text{'}})=C({\rho }_{SB}^{\text{'}})-C({\rho }_{S}^{\text{'}})-C({\rho }_{B}^{\text{'}})$ is called correlated coherence^38,39^.

One can repeat the above procedure to obtain the classical and quantum parts of the entropy production for the new expression, Eq. (16). As was seen before, the entropy production can be divided into two parts,

27 $$\Delta {S}^{irr}=\Delta {S}_{C}^{irr}+\Delta {S}_{Q}^{irr},$$

where

28 $$\Delta {S}_{C}^{irr}=S({\Delta }_{{H}_{S}}({\rho }_{S})||{\rho }_{S}^{eq})-S({\Delta }_{{H}_{S}}({\rho }_{S}^{\text{'}})||{\rho }_{S}^{eq})-S({\Delta }_{{H}_{B}}({\rho }_{B}^{\text{'}})||{\rho }_{B}^{eq})$$

is the classical part, and

29 $$\Delta {S}_{Q}^{irr}=C({\rho }_{S})-C({\rho }_{S}^{\text{'}})-C({\rho }_{B}^{\text{'}})={C}_{cc}({\rho }_{SB}^{\text{'}})$$

is the quantum one. As can be seen, the new definition of heat results in that only the correlated coherence appears in the entropy production expression with no coherence of subsystems, in spite of what is mentioned in ref. ^35^.

Using relative entropy of coherence, one obtains^39^

30 $${C}_{cc}({\rho }_{SB}^{\text{'}})=I({\rho }_{SB}^{\text{'}})-I({\Delta }_{{H}_{S}+{H}_{B}}({\rho }_{SB}^{\text{'}})).$$

Regarding Eq. (30), the entropy production in Eq. (17) can be written as

31 $$\Delta {S}^{irr}=I({\rho }_{SB}^{\text{'}})=[I({\Delta }_{{H}_{S}+{H}_{B}}({\rho }_{SB}^{\text{'}})]+[I({\rho }_{SB}^{\text{'}})-I({\Delta }_{{H}_{S}+{H}_{B}}({\rho }_{SB}^{\text{'}}))],$$

hence, the classical and quantum parts of the new entropy production can be written, respectively, as

32 $$\Delta {S}_{C}^{irr}=S({\Delta }_{{H}_{S}}({\rho }_{S})||{\rho }_{S}^{eq})-S({\Delta }_{{H}_{S}}({\rho }_{S}^{\text{'}})||{\rho }_{S}^{eq})-S({\Delta }_{{H}_{B}}({\rho }_{B}^{\text{'}})||{\rho }_{B}^{eq})=I({\Delta }_{{H}_{S}+{H}_{B}}({\rho }_{SB}^{\text{'}})),$$

and

33 $$\Delta {S}_{Q}^{irr}={C}_{cc}({\rho }_{SB}^{\text{'}})=I({\rho }_{SB}^{\text{'}})-I({\Delta }_{{H}_{S}+{H}_{B}}({\rho }_{SB}^{\text{'}})).$$

Since $I({\Delta }_{{H}_{S}+{H}_{B}}({\rho }_{SB}^{\text{'}}))$ is always non-negative, one comes to $\Delta {S}_{C}^{irr}\ge 0$. $\Delta {S}_{Q}^{irr}$ is also non-negative due to the data-processing inequality related to strong-subadditivity of the von Neumann entropy leading to this fact that mutual information decreases subject to local operations^40,41^. $\Delta {S}_{Q}^{irr}$ can be considered as a discord quantifier which depends on the basis and is established on the concept of local projective measurements detecting the quantumness of correlations^42,43^.

**Figure 1:**
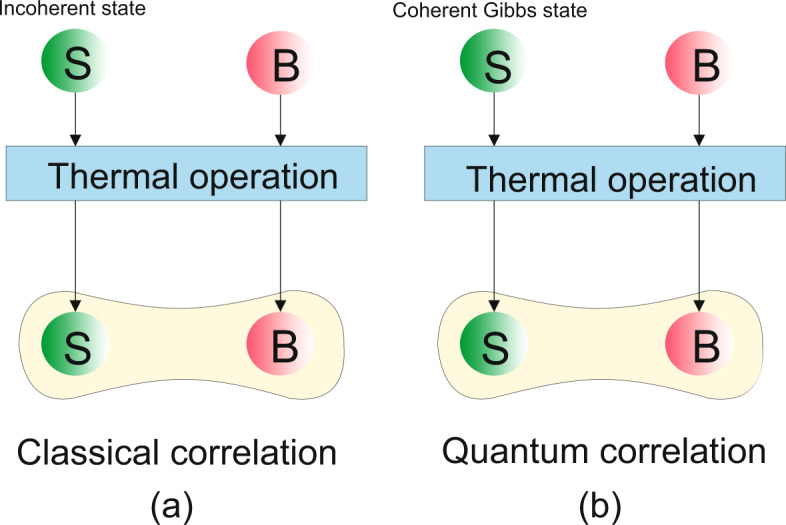
(Color online) (**a**) TOs cannot generate quantum correlation from incoherent input states. (**b**) If input state of the system is coherent Gibbs state, correlation between system and bath is quantum correlation.

It should be mentioned that if the initial state of the system is diagonal in the energy basis, meaning that *C*(*ρ*_*S*_) = 0, one comes to

34 $$C({\rho }_{S}^{\text{'}})=C({\rho }_{B}^{\text{'}})={C}_{cc}({\rho }_{SB}^{\text{'}})=0,$$

which indicates that the system and the bath states remain diagonal subject to time evolution. Also

35 $$\Delta {S}_{Q}^{irr}={C}_{cc}({\rho }_{SB}^{\text{'}})=I({\rho }_{SB}^{\text{'}})-I({\Delta }_{{H}_{S}+{H}_{B}}({\rho }_{SB}^{\text{'}}))=0,$$

therefore

36 $$\Delta {S}^{irr}=\Delta {S}_{C}^{irr}=I({\Delta }_{{H}_{S}+{H}_{B}}({\rho }_{SB}^{\text{'}})),$$

as can be seen, only the classical part appears. Hence, one can say that if the initial state of the system is diagonal in the energy basis, it is impossible to create a quantum correlation between the system and the bath by applying TOs (see Fig. 1). However, if the initial state of the system is the coherent Gibbs state^20,44^,

37 $$|\lambda {\rangle }_{S}:\,=\sum _{i}\sqrt{\frac{{e}^{-\beta {E}_{i}}}{{Z}_{S}}}|{E}_{i}\rangle ,$$

one comes to ${\Delta }_{{H}_{S}}{(|\lambda \rangle }_{S}\langle \lambda |)={\rho }_{S}^{eq}$ and therefore $S({\Delta }_{{H}_{S}}{(|\lambda \rangle }_{S}\langle \lambda |)||{\rho }_{S}^{eq}\mathrm{)=0}$. From Eq. (32) and this fact that $\Delta {S}_{C}^{irr}$ is a non-negative quantity, one can conclude that $\Delta {S}_{C}^{irr}=0$. Hence, for this case, there is no classical part in the entropy production expression,

38 $$\Delta {S}^{irr}=\Delta {S}_{Q}^{irr}={C}_{cc}({\rho }_{SB}^{\text{'}}),$$

which is in contrast to the previous case, Eq. (36), (see Fig. 1). It should be mentioned that the above results are true for the entropy production introduced in Eq. (22); if the initial state of the system is incoherent, one obtains $\Delta {\bar{S}}_{Q}^{irr}=0$, meaning that only the classical part appears in the entropy production expression, and if the system is initially in a coherent Gibbs state, one arrives at $\Delta {\bar{S}}_{C}^{irr}=0$, meaning that only the quantum part is left.

## Example

**Figure 2:**
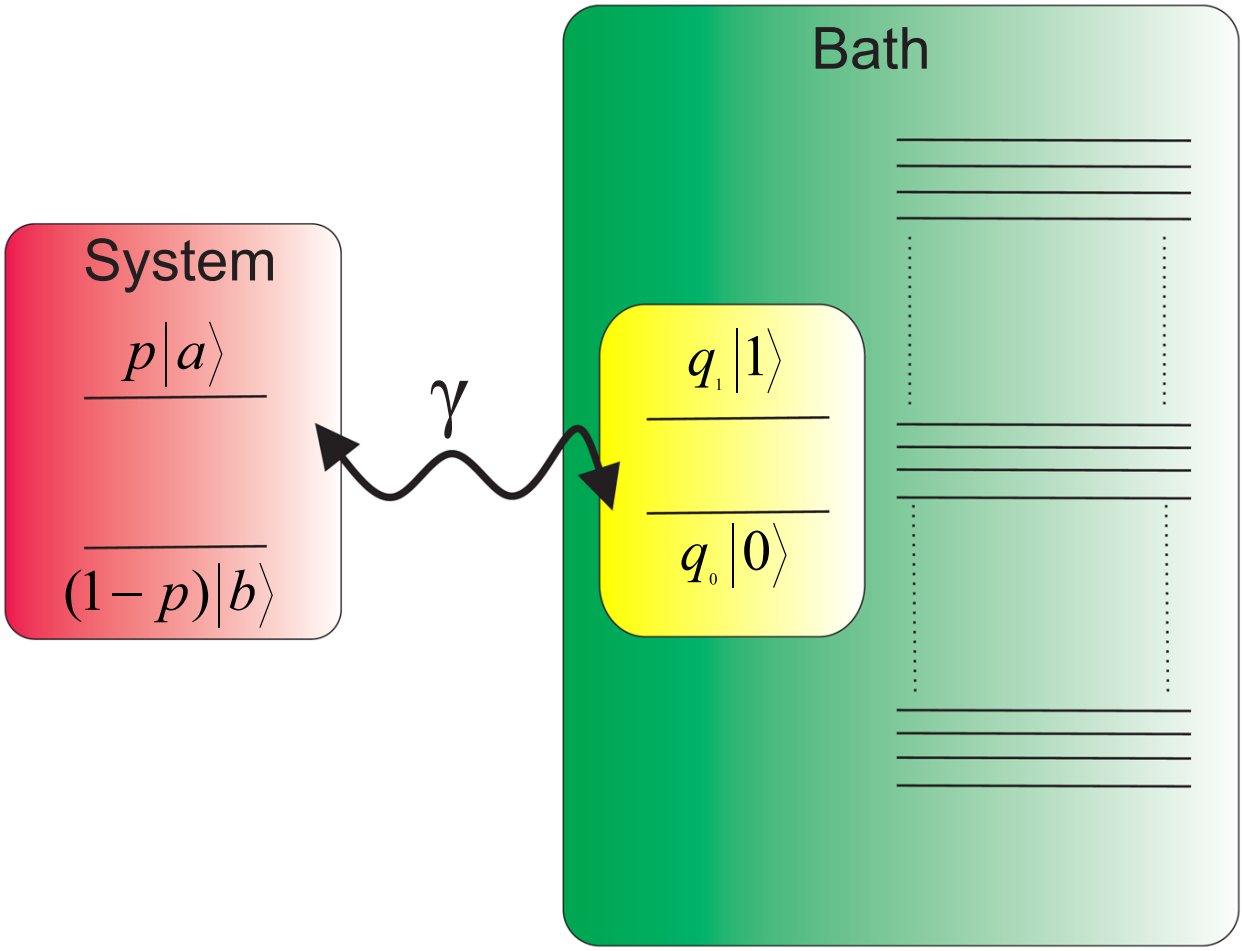
(Color online) Schematic diagram of a bath of virtual qubits. The system is modeled as a two-level system. The bath is in thermal equilibrium at temperature *T* and one can repeatedly pick one virtual qubit out of the bath in each single run of the protocol.

To illustrate the above-mentioned results, let us consider a two-level system whose Hamiltonian is given by ${H}_{S}={\omega }_{a}|a\rangle \langle a|+{\omega }_{b}|b\rangle \langle b|$, where *ω*_*a*_ > *ω*_*b*_ (Fig. 2). The bath is assumed to be a huge reservoir out of which one can freely and repeatedly, in each run of the protocol, pick one copy of a virtual or ancillary two-level system (qubit) which is on resonance with the system^45–47^. The bath Hamiltonian is ${H}_{B}={\omega }_{1}|1\rangle \langle 1|+{\omega }_{0}|0\rangle \langle 0|+{H}_{redu}^{B}$, where *ω*_1_ > *ω*_0_, and ${H}_{redu}^{B}$ is the Hamiltonian describing the dynamical behavior of the rest of the bath. The initial state of the bath is assumed to be a thermal state, therefore, the state of the virtual qubit can be expressed as

39 $${\rho }_{vir}={q}_{1}|1\rangle \langle 1|+{q}_{0}|0\rangle \langle 0|,$$

where ${q}_{i}=\frac{{e}^{-\beta {\omega }_{i}}}{{Z}_{B}}$. Also, the resonance condition is assumed to be

40 $${\omega }_{a}-{\omega }_{b}={\omega }_{1}-{\omega }_{0}.$$

The “thermal contact” between the system and the bath is described by the interaction Hamiltonian,

41 $${H}_{\mathrm{int}}=\gamma (|b\rangle \langle a|\otimes |1\rangle \langle 0|+|a\rangle \langle b|\otimes |0\rangle \langle 1|),$$

where *γ* is coupling strength, and it is assumed that $\hslash =1$. The time evolution of the total system is then governed by the unitary operator ${U}_{t}=exp[\,-\,i({H}_{S}+{H}_{B}+{H}_{int})t]$. After an infinitesimal time *δt* the state of the total system evolves to

42 $${\rho }_{SB}(\delta t)={U}_{\delta t}{\rho }_{SB}(0){U}_{\delta t},$$

where the initial state *ρ*_*SB*_(0) is a direct product of the system and the bath initial states.

**Figure 3:**
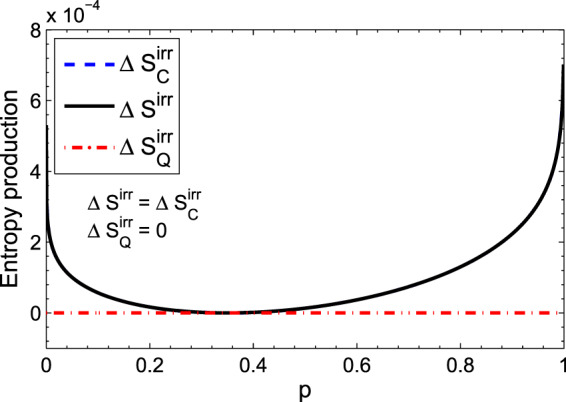
(Color online) (**a**) Variation of the entropy production, its classical and quantum parts with respect to the parameter *p*. The initial state of the system is assumed to be an incoherent state. Numerical values are *q*_1_ = 0.35, *q*_0_ = 0.65 and *γt* = 0.01.

Let us examine the above example for two different initial states of the system. Firstly, the initial state of the system is assumed to be an incoherent state,

43 $${\rho }_{S}(0)=p|a\rangle \langle a|+(1-p)|b\rangle \langle b|,$$

where $0\le p\le 1$. In Fig. 3, entropy production, its classical and quantum parts for this state are plotted versus the parameter *p*. The plots show that the quantum contribution of the entropy production is zero, hence, the entropy production is the same as the classical part. Secondly, let us assume that the initial state of the system is pure and has coherence in the energy basis,

44 $$|\psi {\rangle }_{S}=\sqrt{p}|a\rangle +\sqrt{(1-p)}|b\rangle .$$

**Figure 4:**
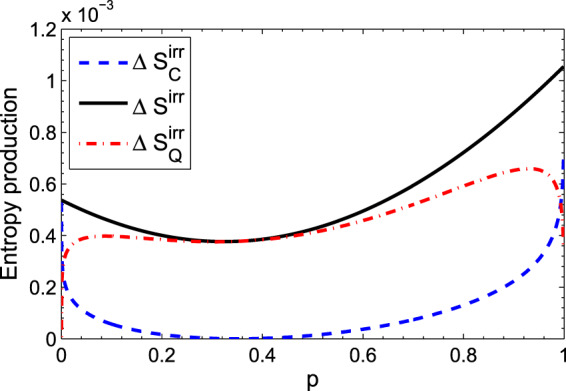
(Color online) The black (solid), the blue (dashed), and the red (dot-dashed) curves represent how entropy production, its classical and quantum parts change over the parameter p, respectively. The initial state of the system is assumed to be $|\psi {\rangle }_{S}=\sqrt{p}|a\rangle +\sqrt{(1-p)}|b\rangle$. Numerical values are *q*_1_ = 0.35, *q*_0_ = 0.65 and *γt* = 0.01.

In Fig. 4, the same three quantities as in Fig. 3 for this state are plotted versus the parameter *p*. As can be seen, when the initial state of the system is the coherent Gibbs state, *p* = 0.35, the classical contribution of the entropy production is zero, which is consistent to what is mentioned before.

### Discussion

In this paper, using the new definition of heat^28^, the corresponding expression for entropy production was obtained. The difference between this expression and the old one is a term which goes to zero in the limit of large baths, meaning that both expressions come close to each other in this limit.

Furthermore, it was shown that the new definition of heat leads to this fact that the entropy production is the same as mutual information between a system and a bath for TOs; it is also true for entropy-preserving operations provided that the initial state of the system-bath is a tensor product state.

It was also shown that the upper bound of the free energy change of a system under TOs is tighter due to this new definition of heat.

Finally, the role of quantum coherence in the new expression of entropy production was studied and it was realized that if the initial state of a system is diagonal in the energy basis, one cannot create a quantum correlation between the system and its bath, subject to TO. On the other hand, it turned out that the correlation between a system and a bath is quantum correlation if the initial state of the system is a coherent Gibbs state.

## Methods

In this section, the derivation of Eqs. (13), (15), (16), (17) and (20) is detailed. The approach is slightly different from the previous methods^22–25^. First of all, it is necessary to note that the following relations are true for TOs:

45 $$\Delta {E}_{S}+\Delta {E}_{B}=\mathrm{0,}$$

46 $$I({\rho }_{SB}^{\text{'}})=\Delta {S}_{S}+\Delta {S}_{B},$$

and

47 $$\Delta {F}_{B}=TS({\rho }_{B}^{\text{'}}||{\rho }_{B}^{eq}\mathrm{)}.$$

Equation (45) is true because of the energy conservation condition, Eq. (46) comes from this condition that the initial total state is a direct product of the system and bath states and the total state evolves unitarily, $S({\rho }_{SB}^{\text{'}})=S({U}_{SB}({\rho }_{S}\otimes {\rho }_{B}^{eq}){U}_{SB}^{\dagger })=S({\rho }_{S})+S({\rho }_{B}^{eq})$, and Eq. (47) is due to the fact that the initial state of the bath is a thermal state.

### Derivation of Eq. (13)

According to Eq. (12), the entropy production is given by

48 $$\Delta {S}^{irr}=\Delta {S}_{S}-\Delta {S}^{rev},$$

in which Δ*S*_*S*_ is the change in the entropy of the system and Δ*S*^*rev*^ is equal to *β*Δ*Q*. Regarding Eq. (8) as the definition of heat, one obtains

49 $$\Delta {\bar{S}}^{irr}=\Delta {S}_{S}-\Delta {S}^{rev}=\Delta {S}_{S}-\beta \Delta \bar{Q}=\Delta {S}_{S}+\beta \Delta {E}_{B},$$

which leads to

50 $$\Delta {\bar{S}}^{irr}=\Delta {S}_{S}-\,\beta \Delta {E}_{S}=-\beta \Delta {F}_{S},$$

due to Eq. (45) and the definition of the free energy. Since $F({\rho }_{S})={F}_{eq}+TS({\rho }_{S}||{\rho }_{S}^{eq})$, one obtains

51 $$\Delta {\bar{S}}^{irr}=-\,\beta \Delta {F}_{S}=S({\rho }_{S}||{\rho }_{S}^{eq})-S({\rho }_{S}^{\text{'}}||{\rho }_{S}^{eq}).$$

### Derivation of Eq. (15)

Substituting Eqs. (45), (46) and (47) into Eq. (50) and keeping in mind the definition of the free energy, one arrives at

52 $$\Delta {\bar{S}}^{irr}=-\,\beta \Delta {F}_{S}=\Delta {S}_{S}-\beta \varDelta {E}_{S}=I({\rho }_{SB}^{\text{'}})-\Delta {S}_{B}+\beta \Delta {E}_{B}=I({\rho }_{SB}^{\text{'}})+\beta \Delta {F}_{B}=I({\rho }_{SB}^{\text{'}})+S({\rho }_{B}^{\text{'}}||{\rho }_{B}^{eq}),$$

which is Eq. (15).

### Derivation of Eq. (16)

The approach is similar to the one used to obtain Eq. (13), the only difference is that here Eq. (9) is considered as the definition of heat. Starting with Eq. (12) and considering the definition of heat according to Eq. (9), one comes to

53 $$\Delta {S}^{irr}=\Delta {S}_{S}-\Delta {S}^{rev}=\Delta {S}_{S}-\beta \Delta Q=\Delta {S}_{S}+\Delta {S}_{B}=\Delta {S}_{S}+\beta \Delta {E}_{B}-\beta \Delta {F}_{B},$$

which, together with Eq. (45) and the definition of the free energy, gives

54 $$\Delta {S}^{irr}=-\beta (\Delta {F}_{S}+\Delta {F}_{B}\mathrm{)}.$$

Now, replacing Δ*F*_*B*_ and Δ*F*_*S*_ from Eqs. (47) and (51), respectively, results in Eq. (16),

55 $$\Delta {S}^{irr}=-\,\beta (\Delta {F}_{S}+\Delta {F}_{B})=S({\rho }_{S}||{\rho }_{S}^{eq})-S({\rho }_{S}^{\text{'}}||{\rho }_{S}^{eq})-S({\rho }_{B}^{\text{'}}||{\rho }_{B}^{eq}\mathrm{)}.$$

### Derivation of Eq. (17)

To obtain Eq. (17), one can use the definition of the free energy and Eqs. (45) and (46) to rewrite Eq. (54) as

56 $$\Delta {S}^{irr}=-\,\beta (\Delta {F}_{S}+\Delta {F}_{B})=\Delta {S}_{S}+\Delta {S}_{B}-\beta (\Delta {E}_{S}+\Delta {E}_{B})=I({\rho }_{SB}^{\text{'}}\mathrm{)}.$$

For entropy-preserving operations ($S({\rho }_{SB}^{\text{'}})=S(\Lambda ({\rho }_{SB}))=S({\rho }_{SB})$), if the initial state of the composite system *SB* is factorized (*ρ*_*SB*_ = *ρ*_*S*_⊗*ρ*_*B*_), one will have

57 $$S({\rho }_{SB}^{\text{'}})=S({\rho }_{SB})=S({\rho }_{S})+S({\rho }_{B}\mathrm{)}.$$

Regarding Eq. (57) and the definition of the mutual information, one obtains $I({\rho }_{SB}^{\text{'}})=\Delta {S}_{S}+\Delta {S}_{B}$, therefore

58 $$\Delta {S}^{irr}=\Delta {S}_{S}-\Delta {S}^{rev}=\Delta {S}_{S}-\beta \Delta Q=\Delta {S}_{S}+\Delta {S}_{B}=I({\rho }_{SB}^{\text{'}}),$$

which is the same as Eq. (56).

### Derivation of Eq. (20)

The free energy for a system in a state *ρ*_*S*_ is

59 $$F({\rho }_{S})=tr({H}_{S}{\rho }_{S})-TS({\rho }_{S}\mathrm{)}.$$

Substituting ${H}_{S}=-\,\frac{1}{\beta }(ln({\rho }_{S}^{eq})+ln({Z}_{S}))$ into the above equation, one obtains

60 $$F({\rho }_{S})={F}_{eq}+TS({\rho }_{S}||{\rho }_{S}^{eq}),$$

where ${F}_{eq}=\frac{-1}{\beta }ln({Z}_{S})$ is the free energy in thermal equilibrium and $S({\rho }_{S}||{\rho }_{S}^{eq})=tr({\rho }_{S}ln({\rho }_{S}))-tr({\rho }_{S}ln({\rho }_{S}^{eq}))$ is quantum relative entropy. Regarding $tr({\rho }_{S}ln({\rho }_{S}^{eq}))=tr({\Delta }_{{H}_{S}}({\rho }_{S})ln({\rho }_{S}^{eq}))$, one comes to

61 $$F({\rho }_{S})={F}_{eq}-TS({\rho }_{S})+TS({\Delta }_{{H}_{S}}({\rho }_{S}))+TS({\Delta }_{{H}_{S}}({\rho }_{S})||{\rho }_{S}^{eq})={F}_{eq}+TS({\Delta }_{{H}_{S}}({\rho }_{S})||{\rho }_{S}^{eq})+TC({\rho }_{S}),$$

where $C({\rho }_{S})=S({\Delta }_{{H}_{S}}({\rho }_{S}))-S({\rho }_{S})$ is relative entropy of coherence.
